# Multimodal FRACTAL-MSI and Image Fusion Improves Detection and Resolution of Biomolecule Imaging

**DOI:** 10.1021/acscentsci.6c00422

**Published:** 2026-07-06

**Authors:** Mika T. Westerhausen, Rosemary J. Bergin, Connor R. Phillips, Jake P. Violi, Thomas E. Lockwood, Reggie Ridlen, David P. Bishop

**Affiliations:** † School of Mathematical and Physical Sciences, 1994 University of Technology Sydney, Ultimo, NSW 2007, Australia; ‡ Hyphenated Mass Spectrometry Laboratory, 1994 University of Technology Sydney, Ultimo NSW 2007, Australia; § Molecular Horizons, 90119 University of Wollongong, Wollongong, NSW 2500, Australia; ∥ School of Life Sciences, Faculty of Science, 1994 University of Technology Sydney, Sydney, NSW 2007, Australia; ⊥ School of Chemistry, 7800 University of New South Wales, Sydney, NSW 2052, Australia; # NanoMicroLab, Institute of Chemistry,University of Graz, Graz 38010, Austria

## Abstract

Elemental mass spectrometry imaging (MSI) of biomolecules via metal-conjugated antibodies enables highly multiplexed, *in situ* quantitative analyses. However, the detection of low abundance analytes by elemental MSI and the spatial resolution of the images obtained are inhibited by detector sensitivity. To overcome this constraint, we introduce FRACTAL-MSI, a signal amplification method that adapts FluoRescent signal Amplification via Cyclic staining of TArget moLecules (FRACTAL) for multimodal laser ablation-inductively coupled plasma-mass spectrometry (LA-ICP-MS) and immunofluorescence (IF) imaging by alternating fluorescent- and lanthanide-tagged secondary antibodies on the same histological section. Using NeuN as a model neuronal marker, we demonstrate 23–80× amplification of IF and LA-ICP-MS signals in fresh-frozen murine brain, FFPE tissue, and SH-SY5Y cells. Two image fusion methods that did not require machine learning were then developed that exploited true probe colocalization to generate quantitative elemental images at IF resolution. The enhanced sensitivity permitted super-resolution reconstruction of LA-ICP-MS data to obtain subcellular images with a 250 nm resolution, or greater than that of the 40× IF obtained via image fusion. We also demonstrated selective target amplification within a 4-plex elemental panel. Together, FRACTAL-MSI provides a practical, broadly accessible strategy to improve detection, and therefore the resolution, of low-abundance biomolecules.

## Graphical abstract

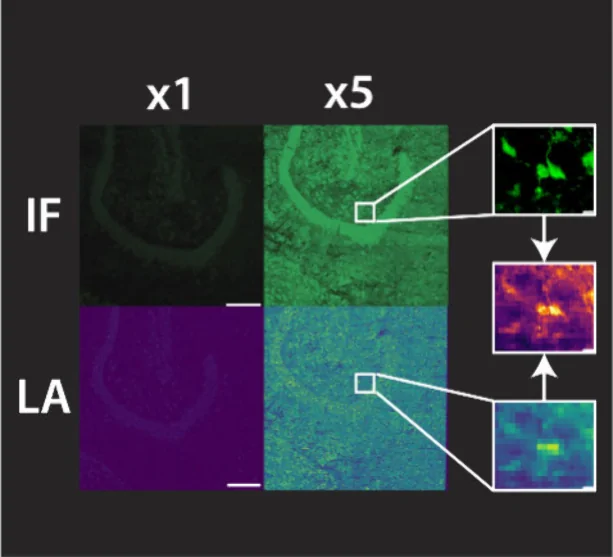

## Introduction

Imaging of biomolecules using elemental mass spectrometry imaging (MSI) and metal-conjugated probes can provide targeted multiplexed protein and mRNA analyses at the resolution required to assign cell types and explore cellular changes and interactions directly in tissue.^1,2^ Compatible techniques for solid sampling of tissues include laser ablation-inductively coupled plasma-mass spectrometry (LA-ICP-MS) and secondary ion mass spectrometry (SIMS),^2^ which include versions of each technology developed specifically for imaging biomolecules such as imaging mass cytometry (IMC)^3^ and MIBI-TOF,^4^ respectively. Elemental MSI can provide simultaneously multiplexed images of up to 40 targets at cellular resolution,^5^ which has led to its use in clinical trials of ocular tissue for the diagnosis of neoplastic and degenerative eye diseases,^6^ the mapping of multicellular dynamics of triple-negative breast cancer patients undergoing treatment,^7^ and guiding clinical treatment of melanomas.^8^

Elemental MSI is advantageous for biological imaging, particularly for highly multiplexed analyses, over techniques such as immunofluorescence (IF) that are hampered by channel mixing or autofluorescence.^9^ However, with elemental MSI now approaching the resolution of IF microscopy, its major limitation is the same historical hurdle: insufficient signal intensity from the detector.^10^ The capacity to determine small changes of biomarker abundance in subcellular regions is fundamental to understanding disease states and their progression; therefore, alternative strategies are required to measure these changes via elemental MSI.

Microscopy-based imaging often uses signal amplification to improve the detection of low-abundance biomarkers. Here, signal amplification may be achieved via increasing analyte visibility (e.g., alter choice of chemical dye or fluorescent analytes to produce signal with stronger intensity)^11^ or increasing the number of analytes by the application of secondary antibodies as theoretically two secondary antibodies bind to each primary antibody.^12^ Fluorescent tyrosine signal amplification uses secondary antibodies to facilitate the deposition of fluorescent tags on surrounding tyrosine residues^13,14^ and can also be used to maximize the signal. These techniques have been used extensively in diagnostics^15^ and have become standard methods for amplification across pathology.^16^

Cyclic signal amplification is another strategy to improve the detection of low-abundance biomolecules. Immuno-SABER (signal amplification by exchange reaction) uses antibodies conjugated with fluorophore-tagged ssDNA concatemers to directly amplify the signal from each antibody. Connection-type ssDNA can also be used to create multiple branches off the originally conjugated concatemer, with each branch then able to bind additional fluorophore-tagged ssDNA to exponentially increase amplification.^17^ This method has been modified to contain metal analytes for detection by IMC (SABER-IMC)^18^ and for X-ray fluorescence imaging.^19^ The shortcoming of this method is its overall complexity. Concatemers (either branched or linear) need to be designed, manufactured, and validated for each antibody you wish to use as the primary probe.^20^ The concatemers are then bound to the primary antibodies, followed by conjugation of the DNA with the analyte of choice (i.e., fluorescent or mass tag) before it is applied to the tissue. Despite their near limitless customization potential, the time and monetary costs associated can be prohibitive. An alternative cyclic signal amplification strategy with lower complexity is FluoRescent signal Amplification via Cyclic staining of TArget moLecules (FRACTAL).^21^ FRACTAL uses two commercially available fluorophore-tagged secondary antibodies chosen for their cross-reactivity, which are then bound iteratively to form a selected number of layers. The multiple layers of secondary antibodies result in amplification of the immunofluorescent signal; however, the number of amplified multiplexable targets is restricted by the viable combinations of host species (e.g., donkey anti-rabbit and rabbit anti-donkey) used to manufacture secondary antibodies.^22^

An approach to improving MSI resolution is to fuse lower resolution MSI images with higher resolution IF images. Image fusion is common in hyperspectral imaging.^23^ Molecular MSI techniques such as matrix-assisted laser desorption/ionization (MALDI)^24–27^ and desorption electrospray ionization (DESI)^26–29^ have adopted image fusion as they are nondestructive, and post-MSI optical imaging with higher resolution is common for tissue morphology analysis via hematoxylin and eosin (H&E) staining. There are only limited examples of image fusion with LA-ICP-MS data, and they include the use of neural networks to improve the resolution of elemental images using brightfield and autofluorescence microscopy images taken prior to ablation.^30^ The image fusions used in all of these examples have the same underlying assumption, that the distribution of MSI data closely follows that of the second image, typically a micrograph of tissue morphology. For images with large differences in resolution, where image fusion is most effective, it is not possible to prove that this assumption is correct. This often results in visually observable differences in the distribution of the fused image compared with that of the original MSI image.^23,31^ The outcomes of these fusions are images that do not improve sensitivity and are purely qualitative. Neural nets have been used to reduce incorrect predictions but require a large training data set to be effective. Therefore, a more direct image fusion technique without machine learning prediction would be preferred. Additionally, most of these multimodal approaches use consecutive or adjacent sections for different imaging types, which can confuse algorithms^32^ and hamper preprocessing due to the lack of pixel-to-pixel registration.^33^

Here we present an innovative approach to increase sensitivity and resolution of elemental MSI by adapting FRACTAL with secondary antibodies that were alternatively tagged with fluorescent and metal analytes for multimodal IF and LA-ICP-MS imaging, which we have labeled as FRACTAL-MSI. The biomarker NeuN was used as a model target to demonstrate FRACTAL-MSI as it is commonly used in neurodegenerative research where its low expression is a sign of neurological damage; therefore, it is a prime target for amplification.^34^ Two pixel-to-pixel image fusion approaches that did not require machine learning were developed, and the new workflow was used to correlate and quantify NeuN in a single murine brain section at the resolution of the IF. As the analytes used for both images are directly colocalized from the same section, no assumptions about distribution similarity are required. Furthermore, we show that the signal amplification of the elemental label through this approach improved detection sensitivity, allowing higher-resolution elemental MSI images to be obtained.

## Results

### Multimodal IF and Elemental MSI

We first examined the feasibility of incorporating a metal-labeled secondary antibody into the FRACTAL process of applying iterative layers of secondary antibodies. In the following experiments, each generation, or G-layer, contains one incubation of the FITC-conjugated secondary antibody, followed by an incubation of the metal-conjugated secondary antibody. Therefore, at the G1 level, each primary antibody will bind two of the secondary antibodies applied first, in this case the FITC-conjugated secondary, and four of the secondary antibodies applied second, here the metal-conjugated secondary (Figure 1). This is not a linear relationship, and as shown in Table 1, the G5 level theoretically contained 682 fluorescent analytes and 1364 elemental analytes. However, because of steric hindrance, it is not expected that this theoretical maximum will be achieved at the higher G levels.^21^

**Figure 1:**
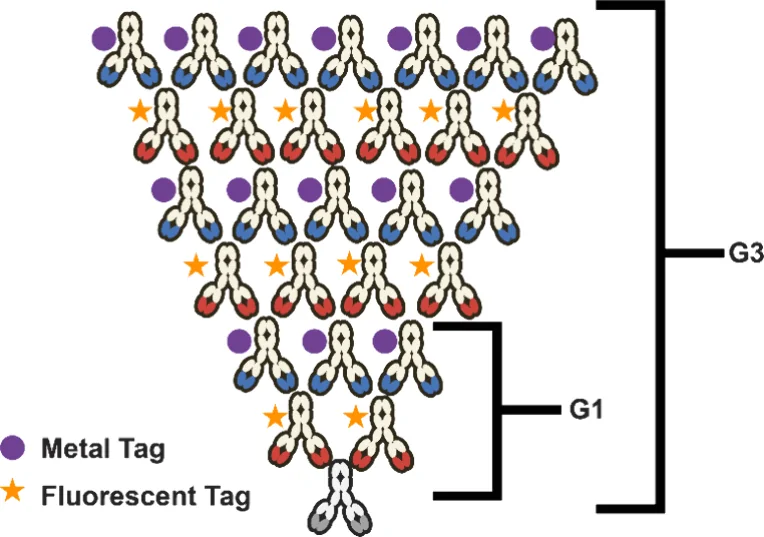
Illustration of G1 to G3 antibody layers. After application of the primary anti-NeuN antibody (gray), the fluorescent-labeled secondary antibody that has affinity with the primary antibody was applied (red), followed by the metal-labeled tertiary antibody (blue) that was chosen to recognize the fluorescent secondary antibody. In each layer, 2 secondary antibodies bind to a single antibody in the layer underneath, highlighting the nonlinear nature of the signal amplification.

The results of this experiment are shown in Figure 2. The IF images were obtained at 40× magnification (Figure 2a–e) and the elemental MSI at 1 μm resolution (Figure 2f–j). All images are set on the same IF or elemental MSI scale for direct comparison. The nonlinear relationship described above is apparent. There are smaller degrees of enhancement between G1 and G2, before a larger jump between G2 and G3. The fluorescent intensity started to plateau at G4, while the increases in metal concentration is smaller between G4 and G5 than between G3 and G4. There is good agreement in the degree of amplification between the IF and the elemental MSI images at the different levels apart from level G3 (Figure 2c,h), where a greater variance is detected. This may be due to blinking and bleaching of the fluorophores,^35^ the nonlinearity of the fluorescent camera response^36^ (as G3 was used to benchmark the image color scales of the other G-levels) and/or comparisons against the larger linear dynamic range and quantitative capabilities of LA-ICP-MS. The results seen here are in good agreement with the original FRACTAL method, where they found a reduction in the degree of signal amplification after 6 iterations of their fluorescent tag.^21^ Due to the addition of the metal-conjugated antibody, that is equivalent to the G3 level here.

**Figure 2:**
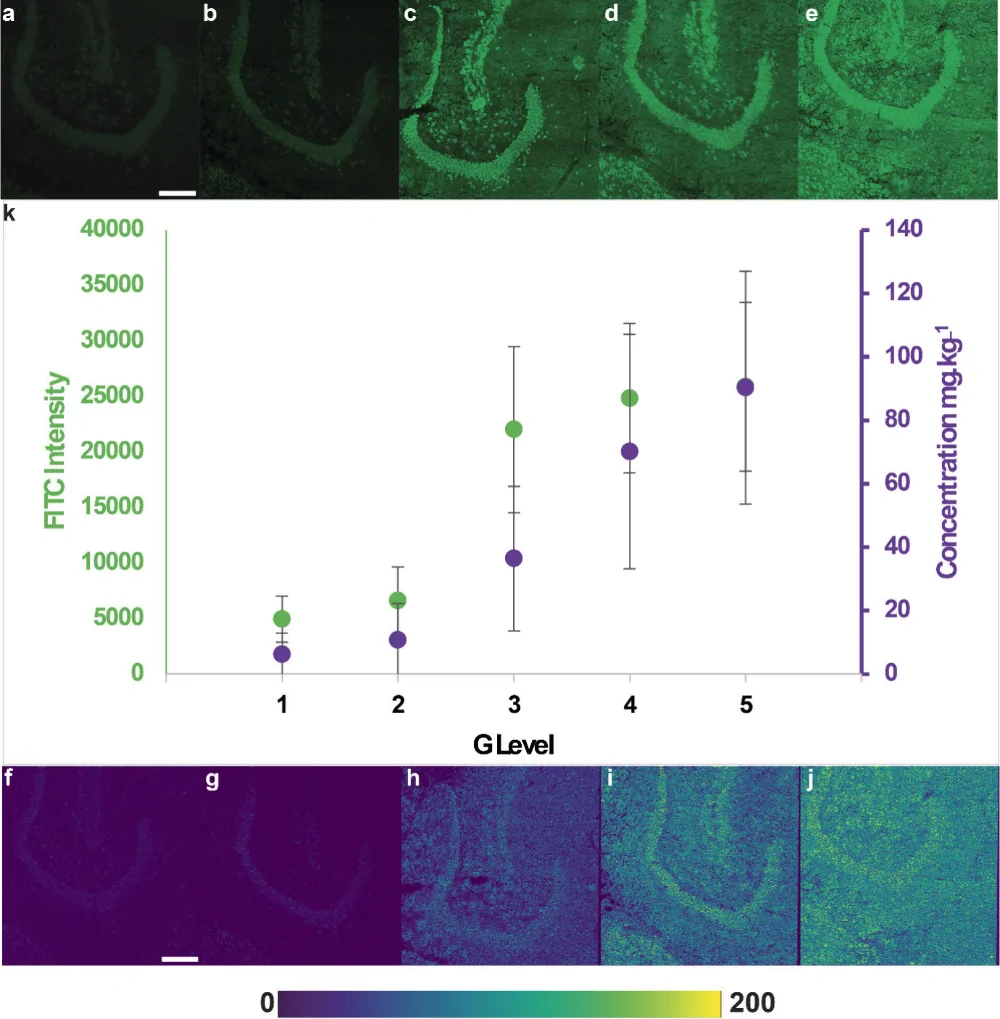
Comparison of G1 to G5 level fluorescence intensity and metal concentration. Images a–e are G1 to G5 fluorescent images of FITC at 40× magnification, and f–j are the corresponding elemental images of ^169^Tm at 1 μm spot size. All images are scaled to the G3 sample (c and h for IF and LA-ICP-MS, respectively). The central scatter plot (k) displays the average image intensity of the FITC compared to the ^169^Tm concentration and their standard deviation as error bars. The scale bar is 200 μm. The calibration bar is in mg kg^–1^. The counts per second values for the calibrated LA-ICP-MS images are provided in Table S1.

### Image Fusion

Above we demonstrated the successful amplification of both fluorescent and elemental signal proportional to their staining level, overcoming the lack of detector response to low abundance analytes. However, the resolution of the images obtained by quantitative elemental MSI is still lower than that of IF images, and ablation at a 1 μm resolution is a relatively slow process. Figure 3a shows a LA-ICP-MS image of neurons in the hippocampal horn of a murine brain acquired at 5 μm resolution to speed up the imaging process, and the corresponding IF image obtained using a 40× objective, equivalent to 0.33 μm resolution, is shown in Figure S1. A region of interest with distinct cells was identified (Figure 3b), where it is evident in the IF image (Figure S1) that what appears to be a single cell in the center of the LA-ICP-MS image is two cells side-by-side. Therefore, we explored two image fusion approaches to exploit the colocalization of the fluorescent and elemental tags, enhancing resolution of the LA-ICP-MS image while maintaining the quantitative nature of the data.

**Figure 3:**
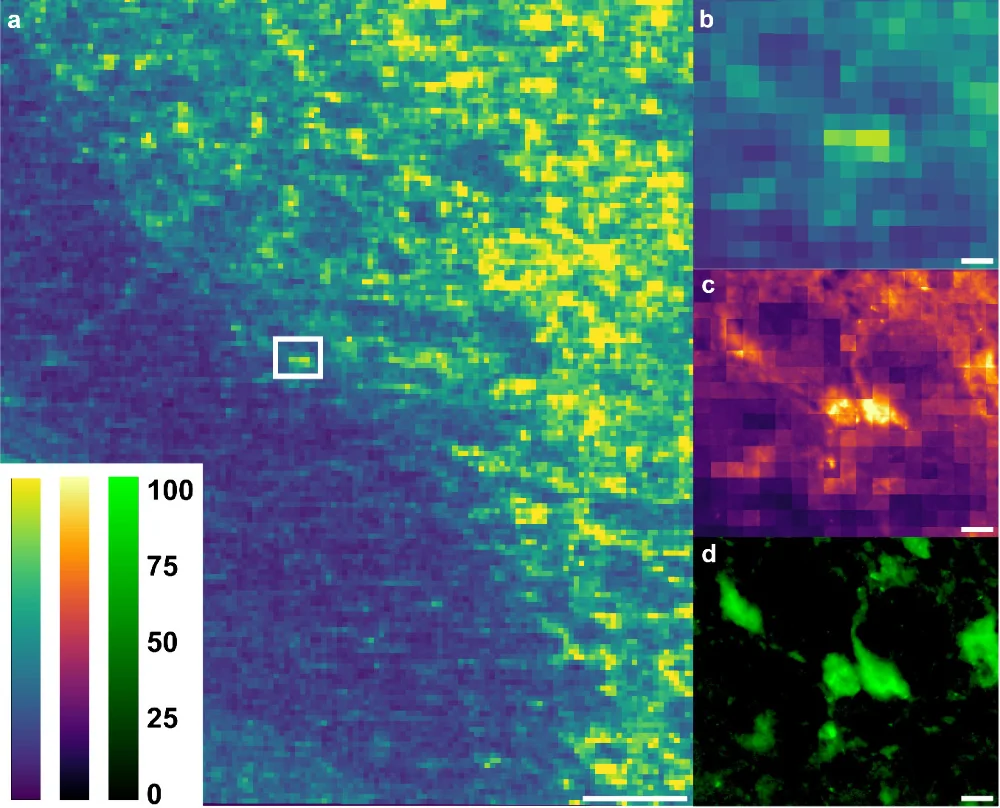
Illustration of the resolution improvements an unprocessed 5 μm resolution LA-ICP-MS image gains from the two image fusion methods. a) LA-ICP-MS image of NeuN in murine brain after amplification to G5, registered to the corresponding 40× fluorescent image, b) square region inset of a neuron from the LA-ICP-MS image, c) PRIF of the inset identifies two neurons are present, and d) CDFIF adoption of the IF image confirms two neurons. The scale bar of a is 200 μm and b–d is 10 μm, and the calibration bar is in mg kg^–1^.

The results of the two image fusion approaches are shown in Figure 3c,d. The resolution improvements on the LA-ICP-MS image is pronounced and below the laser ablation beam width with both approaches. The first approach, pixel redistribution image fusion (PRIF, Figure 3c), split each LA-ICP-MS pixel into the equivalent amount of IF pixels and redistributed the LA-ICP-MS pixel’s concentration evenly across the positive signal pixels from the IF. Each 5 μm LA-ICP-MS pixel is equivalent to more than 1000 IF pixels (0.146 μm^2^ pixels at 0.33 μm resolution) the sum of which are made equivalent to the signal from the single LA-ICP-MS pixel by sum normalization. The second approach, cumulative density function image fusion (CDFIF, Figure 3d), used the intensity histograms of both images to normalize the distribution of the IF image to the quantified LA-ICP-MS image, thereby the IF color scale adopts the quantitative mg kg^–1^ units of the LA-ICP-MS (Figure S2). Both of these methods of image fusion are the first MSI methods to retain quantitative information after processing. With this in mind, CDFIF will be used as a proxy for immunofluorescent images from here on so that color intensity can be compared directly to unprocessed LA-ICP-MS images, and to PRIF, as the only difference between IF and CDFIF is the intensity range (see Figure S3).

We then applied this process to the analysis of cells in a culture. Biomolecules in cells are often difficult to analyze by IF and LA-ICP-MS due to their small size and lower epitope levels compared to tissue per volume.^37^ Comparison between normal staining levels (G1) and amplified staining (G5) on cell culture samples was performed to compare target concentrations. The G1 signal was just above the background noise (see Figure S4a); however, the G5 sample had a quantifiable ^169^Tm signal. Due to the increased signal obtained with the amplification and the small size of the cells requiring smaller areas of ablation, we improved the resolution of the LA-ICP-MS image from 5 to 1 μm for the analysis of the remaining samples.

SH-SY5Y human neuroblastoma cells (Figure 4) were used as they naturally express NeuN, and when differentiated become neuron-like and are used as proxy neurons for studying neurodegenerative diseases such as Parkinson’s disease.^38^ Both the normal and differentiated cells had a similar average concentration; however, the differentiated cells show localized regions of high NeuN abundance (Figure 4e,f). This highly abundant region was also evident in the corresponding CDFIF image (Figure 4h) and has been previously observed by IF.^39^ These images show the limitation of PRIF (Figures 4c,g). As the pixel sizes between LA-ICP-MS (Figure 4b,f) and IF (Figure 4d,h) get closer, the fidelity improvement is reduced compared to that observed with the 5 μm resolution LA-ICP-MS image shown in Figure 3. This is expected as less IF pixels (roughly one-twentieth, e.g., 47 IF pixels/1 μm LA-ICP-MS pixel) are available to make image fusion improvements, and by extensions shows the high fidelity of the 1 μm LA-ICP-MS image. Therefore, the improvements in LA-ICP-MS image resolution enabled by the amplification of the signal decrease the need for image fusion with NeuN as the target in cells.

**Figure 4:**
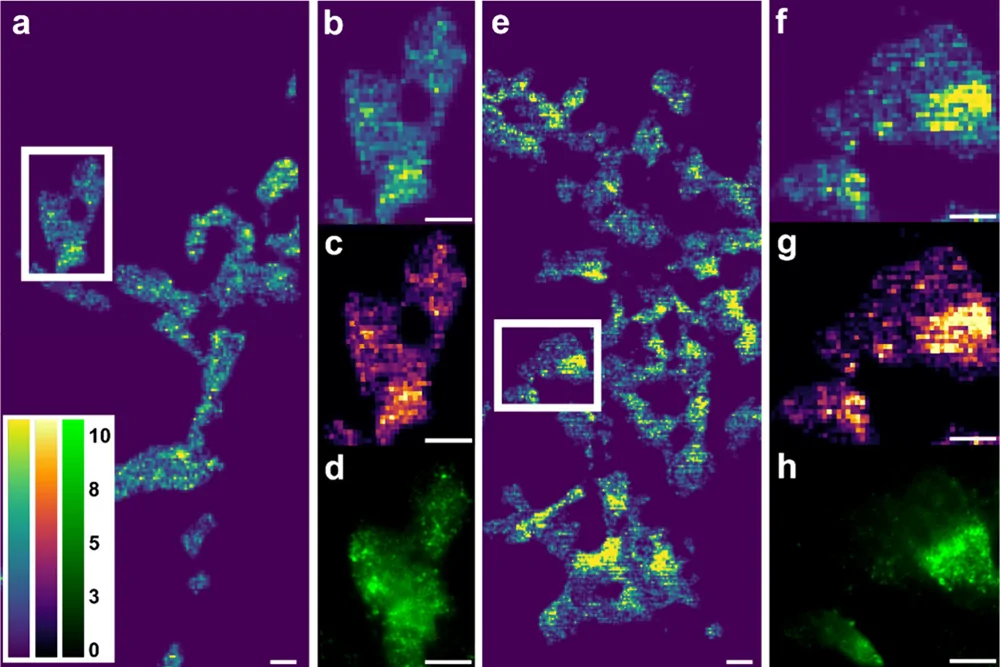
Subcellular resolution imaging of NeuN in SH-SY5Y cells amplified to G5, using 1 μm resolution LA-ICP-MS and image fusion. a–d are standard SH-SY5Y cells, and e–h are the differentiated cells. a,e) Selection of individual cells for image fusion (shown in b,f), PRIF of selected cells (c,g), and CDFIF of selected cells (d,h). The scale bar is 10 μm, and the calibration bar is in mg kg^–1^.

FFPE tissues are a sample type where detection of biomolecules can be problematic as the FFPE process may mask the natural abundance of epitopes, even after antigen retrieval.^40^ Therefore, we examined the capacity of FRACTAL-MSI to improve imaging in FFPE tissues. Similar to the cell culture sample above, G1 was applied as a comparison between normal staining levels, and G5 for amplification with imaging at 1 μm LA-ICP-MS resolution. The results are shown in Figure 5. Image fusion was not applied to the FFPE samples due to the increased autofluorescence artifacts in FFPE tissue^41^ reducing the accuracy of the image fusion. DAPI was added as a counterstain to characterize the tissue, and as a target to focus on during fluorescence imaging since the G1 stain was anticipated to be low and insufficient for clear imaging. Neither the IF image or that obtained by LA-ICP-MS showed a positive response to NeuN on the FFPE tissue at G1 at the intensity scale matched to G5 (Figure 5a,b). Some autofluorescence artifacts and DAPI serve as an intensity benchmark and focal target in the IF image. Application of anti-NeuN antibodies to FFPE tissue after antigen retrieval is known to produce little to no signal when imaging via IF, with alternative neuronal epitopes normally targeted as a result.^40,42^ The neurons are, however, clearly observed after amplification to G5 in the fluorescent and LA-ICP-MS images (Figure 5c,d), indicating that this lack of signal at G1 was due to the low epitope abundance in FFPE tissue described above. Figure 5 panels b and d also demonstrate an advantage of images obtained by elemental MSI over IF, with a region of autofluorescence observed in IF not impacting the LA-ICP-MS images.

**Figure 5:**
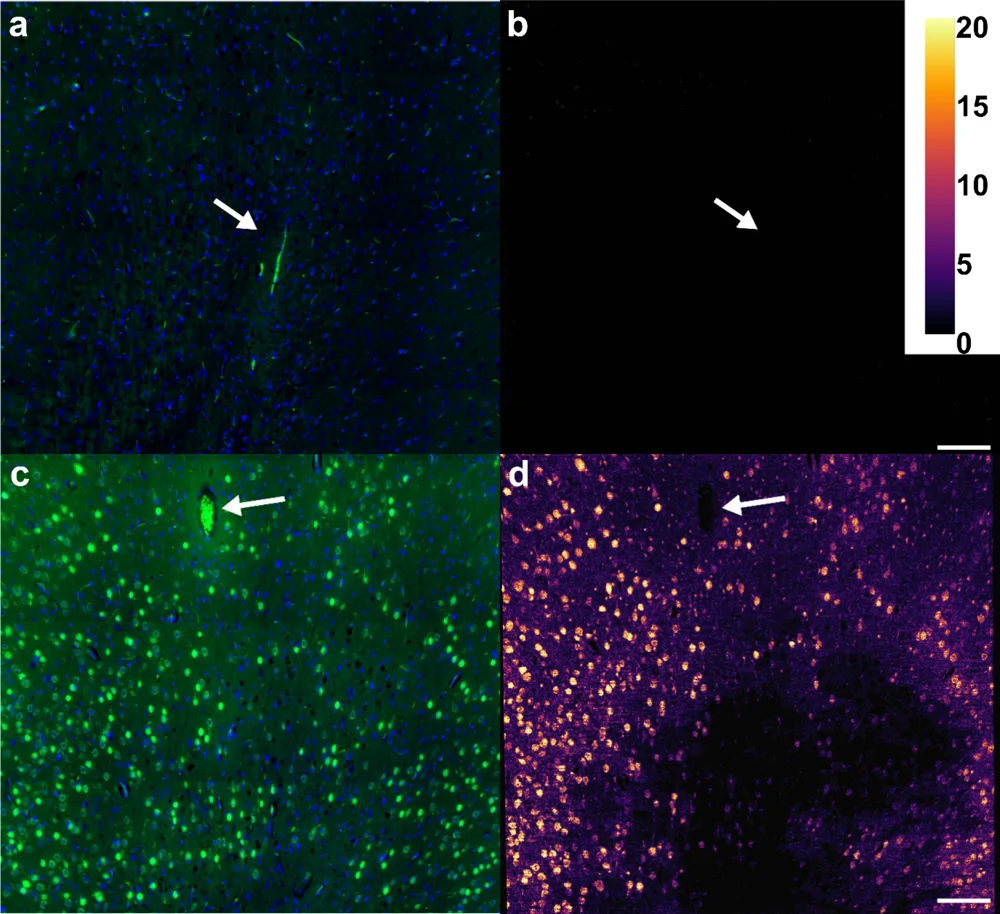
Two-color fluorescent images of FITC (green) and DAPI (blue) at G1 (a) and G5 (c) staining of NeuN, and their respective unprocessed 1 μm resolution LA-ICP-MS images at G1 (b) and G5 (d). White arrows denote suspected autofluorescent artifacts, and the corresponding location where no artifacts are observed in the LA-ICP-MS image. The scale bar is 100 μm, and the calibration bar is in mg kg^–1^. A rescaled version of Figure 5b is available in Figure S4b to show that antibody binding was successful just at a signal level that could not be detected via IF, or illustrated on the same scale as the G5 LA-ICP-MS image.

### Nanoscale Imaging via Super-resolution Reconstruction

Here, given the increased detection sensitivity provided by FRACTAL-MSI, we applied super resolution reconstruction (SRR) to obtain a quantified LA-ICP-MS image with a resolution of 250 nm. The results are shown in Figure 6. Figure 6a shows the 40× IF image, which provides a resolution of 330 nm, Figure 6b is the LA-ICP-MS image, and Figure 6c the overlaid IF and LA-ICP-MS image. Figure 6c shows agreement between the two imaging modalities and that the improved resolution obtained by SRR over IF results in less image spread. There are some minor discrepancies between the two images, with weaker fluorescing cells identified in the IF image not showing the corresponding LA-ICP-MS image. This is not unexpected, as the cells may not be evenly distributed through the depth of the tissue section,^43^ and the summed multilayer SRR process can result in reduced signal if the cell is only present in one of the layers. It is also evident that the SRR image contains more noise than the IF image.

**Figure 6:**
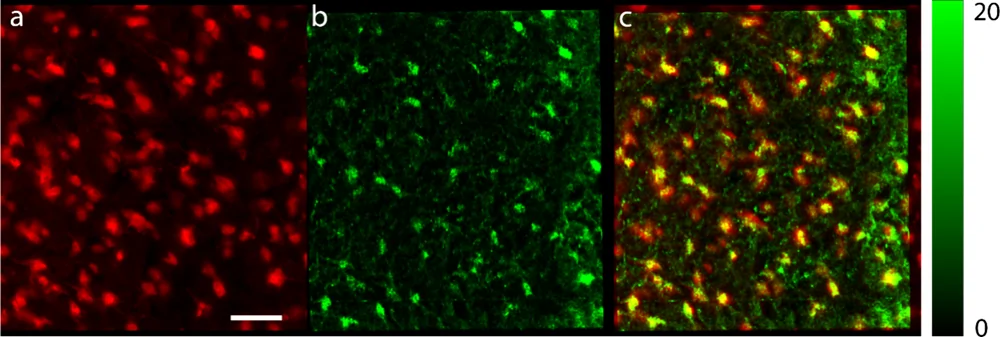
Comparison of submicron imaging NeuN stained at G3. a) 40× IF of FITC is colored red, b) SRR of LA-ICP-MS produces an image with a resolution of 250 nm, and c) the registered overlay of the two channels, showing agreement between the imaging modalities. The scale bar is 20 μm, and the calibration bar is mg kg^–1^.

### Application in a Multiplexed Workflow

Finally, we examined if the signal amplification process could be applied into a multiplexed analysis of biomolecules by LA-ICP-MS. Here we selected CD45 and MMP-11 as additional targets, which required careful selection of anti-MMP-11 and anti-CD45 primary antibodies that did not have cross-reactivity with the immunofluorescent or elemental-tagged secondary antibodies (i.e., the sample tissue is mouse and the primary antibody host is rabbit; therefore, the secondary and tertiary antibodies require alternate hosts^22^). Additionally, we included a DNA intercalator as a marker for cell nuclei. DNA intercalators are commonly added for cell segmentation purposes in elemental MSI and work as a proxy for counterstains such as DAPI.^44^ The results are shown in Figure 7. CD45 is a transmembrane protein with a large extracellular domain^45^ and MMP-11 is an intracellular enzyme. Both colocalize with NeuN as expected in a normal adult mouse brain.^46^ Image fusion was performed on NeuN and is shown in Figure S5, but the large difference in resolution resulted in a multicolor image that was difficult to interpret. Additionally, a high resolution 1 μm spot image was attempted; however, the signals for MMP-11, CD45, and the Ir-intercalator were too low to quantify, and therefore a 5 μm spot was used here. Multielemental imaging by LA-ICP-MS requires a reduction in the per ion integration time when using a quadrupole ICP-MS, and by extension the raw signal obtained for each element. The 4-color multiplex LA-ICP-MS image displayed here shows that FRACTAL-MSI is compatible with a multiplexed workflow, and the use of alternative elemental mass spectrometers such as ICP-TOF or MIBI-TOF may allow higher resolution imaging.

**Figure 7:**
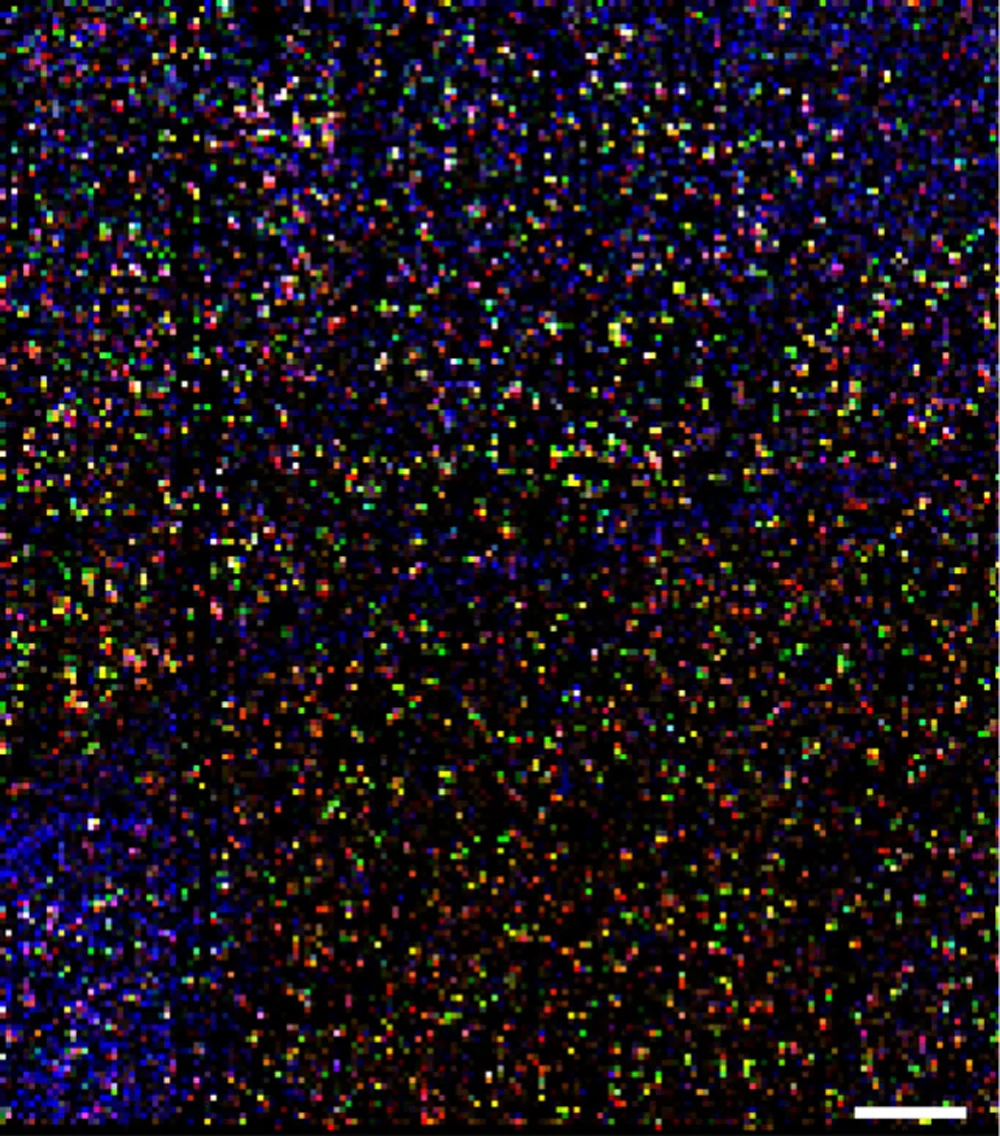
Five μm resolution multiplex false color image of the nuclei (^193^Ir intercalator, blue), NeuN (^169^Tm, green), CD45 (^170^Er, red) and MMP-11 (^142^Nd, Orange). The corresponding individual channels are found in Figure S6. The scale bar is 100 μm.

## Discussion

Here, we developed a multimodal IF and LA-ICP-MS imaging protocol on single histological sections to amplify signal and to aid machine-learning-free image fusion, which was applicable to fresh frozen tissue, fixed cells, and FPPE tissue. The method is flexible and simple to implement, and all of the reagents are commercially available. Additionally, the approach can be modified to amplify multiple targets,^22^ or the analytes could be altered for different imaging modalities. The majority of applications that report dual LA-ICP-MS/microscopy methods were not suitable for image fusion due to restrictions in the imaging modalities used (e.g., brightfield microscopy),^30,47,48^ the use of dyes or probes like lectins^49^ that have nonspecific binding and are not self-binding like antibodies, or are instances where the two imaging modalities targeted different biomarkers.^50^ The combination of LA-ICP-MS with fluorescence microscopy has been applied to analyze multimodal analytes and includes the use of fluorescent nanoclusters^51–53^ and metal-containing fluorescent dyes.^54,55^ However, these multimodal analytes were typically used for complementary imaging, with IF often preferred for antibody optimization due to rapid imaging when compared to LA-ICP-MS.

We demonstrated the degree of amplification obtained across five “generations” (G-levels) of the iterative application of fluorescent- and metal-tagged secondary antibodies. Using the maximum ^169^Tm concentrations obtained to compare the degree of amplification between the G1 and the G5 levels, the values were 30× greater on fresh-frozen tissue, 83× greater in the cells, and 23× on the FFPE tissue. G1 is still amplified compared to directly tagging a primary antibody as the metal-conjugated secondary antibody was applied after the fluorescent-tagged secondary; therefore, it contains 4 secondary antibodies and, as such, these values are a conservative estimate of amplification over a primary-conjugated antibody. Taking into consideration the time taken for staining, the reagent costs, and the amount of amplification gained, the G3 level is suitable for general use. The FRACTAL-MSI protocol also resulted in a method that detected NeuN in FFPE samples where it was thought refractory by immunolabeling methods. This suggests that the amplification overcomes epitopes hidden by the FFPE process and could therefore be used to interrogate other epitopes that are naturally low in abundance^56,57^ and to maximize panels for multiplexed imaging. Signal amplification would also be beneficial during same-slide multiomics, where repeated interrogation of the sample by different imaging modalities causes signal reduction downstream in the imaging workflow.^1^

Only a few examples of signal amplification for LA-ICP-MS have been previously described. When compared to FRACTAL-MSI, SABER-IMC was qualitative,^58^ and the addition of N_2_ to the plasma, while beneficial as it is a uniform, nontargeted method of signal amplification, only obtained modest results with an average 2× amplification of lanthanides.^59^ Solution ICP-MS-based methods of signal amplification include the use of gold nanoparticle tyramide signal amplification^60^ and up-conversion nanoparticle assemblies.^61^ These methods have the potential to obtain similar degrees of amplification and multimodality that could be used for imaging and image fusion.

This is the first example of image fusion being used to improve the resolution of quantified LA-ICP-MS images. LA-ICP-MS images obtained at 5 μm resolution were fused with IF images, resulting in theoretical improvements of 15× when the 5 μm spot was used, and 3× for the 1 μm spot. The respective advantages and disadvantages of the two image-fusion approaches, PRIF and CDFIF, directs their future use. PRIF applies the concentration of the larger LA-ICP-MS pixel to the underlying IF pixels and is suitable for determining concentrations in specific regions of interest where the additional resolution can highlight features. Disadvantages of PRIF include the necessity of accurate registration of the two images, which is similar to existing image fusion approaches, and the increase in image noise reducing image quality and signal-to-noise. The sources of image noise for LA-ICP-MS and IF include electrical noise of the detectors, fluctuations in the electrical current, or detector mis-triggering (e.g., γ rays). This is evidenced in Figure 3c, which displays a reduction from the theoretical image resolution of 330 nm (see Figure 3d). CDFIF adopts the image data of the IF image, therefore providing higher resolution images but also inheriting the flaws of IF. Both methods are affected by artifacts from either imaging modality such as high spike pixels in the LA-ICP-MS, or autofluorescence in the IF (both localized autofluorescence or broadly located across a sample (see Figures S7). However, the overall quantitative characteristics of the fused image is different. Local quantities are more accurate with PRIF, but any artifacts affect the global mean concentration. Whereas with the CDFIF, the global mean remains unchanged, for the specific regions affected by autofluorescence, the local values would be less accurate. As the G-levels increases, the enhanced signal clarity leads to a closer spatial match between the CDFIF image and the LA-ICP-MS, thus reducing the negative effects of fluorescent artifacts.

Image fusion methods that use machine learning are generally unsuitable for exploratory studies with smaller sample cohorts as the predictions have lower accuracy from restricted training sets.^62^ The machine learning-free image fusion approaches we presented here are not impacted by smaller sample sizes; however, they do require compatible probes for each imaging modality such as AmberGen’s Photocleavable Mass Tag (PCMT) technology^63^ that contains fluorophores alongside the peptide mass tags for MALDI-MSI. As LA-ICP-MS is destructive and therefore a final analysis step, other MSI methods that are less destructive such as MALDI-MSI or DESI-MSI^64^ could potentially be incorporated into this workflow,^65^ increasing the information obtainable from a single histological section. Analytes would be required for each imaging modality for cross target normalization and registration before image fusion.^66^

Similar to amplification, only limited examples of image fusion or registration with LA-ICP-MS as one of the modalities have been described. The only example of image fusion to improve image resolution with qualitative LA-ICP-MS improved their resolution 10-fold from 50 to 5 μm.^30^ Their approach used brightfield and autofluorescence microscopy to identify correlated structure between LA-ICP-MS images of zinc and iron. They had to carefully select features that were visible in both the optical and LA-ICP-MS training data to maximize the accuracy of their machine learning model, which is not suitable for smaller data sets. LA-ICP-MS images have been registered with other modalities, but for classification or segmentation, not image improvement.^67–69^

To the best of the authors knowledge, this is the first quantified LA-ICP-MS method for the analysis of biomolecules at 1 μm resolution in single cells and tissue after the application of metal-conjugated antibodies. Other single cell LA-ICP-MS methods use a single laser spot to ablate the whole cell in a single shot,^70^ or those that image at comparable resolution are reported as count per second values.^71^ Using the amplification method developed here, we are imaging at the smallest spot size of commercially available laser ablation instruments, obtaining a signal well above the signal-to-noise at cellular resolution. We have overcome the limitation of lack of detector sensitivity; therefore, this method will be applicable to obtaining subcellular resolution if future instrumental developments enable imaging with spot sizes lower than 1 μm. Unlike with reflective and transmissive imaging, LA-ICP-MS imaging resolution is equivalent to the stage movement between sampling pulses. This unique attribute and the increased detection sensitivity also allowed us to apply an alternative technique, super resolution reconstruction, to improve resolution to 250 nm.^72^ SRR uses oversampling and deconvolution, and when these approaches were first attempted, the resolution obtained was limited by the technology available at the time, with image resolution improvements from 5.0 × 5.0 μm to 0.5 × 1.0 μm^73^ and 15 μm to ∼3.6 μm.^74^ A recent demonstration of SRR improved image resolution from 1 μm to 350 nm^75^ or similar to a 40× IF image. Their spot-wise imaging protocol differed from the raster ablation applied here, which allowed us to image below the resolution of 40× IF to 250 nm.

The feasibility of including FRACTAL-MSI into a multiplexed analysis to selectively amplify a target was investigated. Two primary-conjugated antibodies and a DNA intercalator were added to create a 4-plex analyte list and were imaged and quantified with a 5 μm spot size. Higher resolution images were unable to be obtained, with smaller spot sizes not producing enough signal for the unamplified targets, and image fusion highlighted difficulties when trying to visualize targets in a single image with large differences in resolution. The potential of image fusion for multiplexed analysis is apparent but requires each channel to have their own metal and fluorescent tag pair to make best use of resolution improvement. Alternate instrumentation may also allow reduced laser spot sizes to be used for analysis.^5,10,76^

FRACTAL-MSI does contain limitations. Quantification is relative, as the combination of the amplification process and the heterogeneous antibody-metal tag conjugation process^77^ result in an unknown number of metal atoms per biomolecule, knowledge of which is required for absolute quantification. However, as all samples are treated equally, and analyzed alongside external calibration standards, the relative concentrations obtained are comparable. Additionally, the majority of quantified elemental MSI methods would be considered to use relative quantification.^2^ Multiplexing via FRACTAL-MSI is limited to just 4 epitopes due to cross-reactivity between antibody host species.^22^ However, we have shown here that FRACTAL-MSI can be applied in conjunction with primary antibodies conjugated with metal analytes, allowing a multiplexed analysis with amplification of specific targets. The level of amplification achievable for increased multiplexing by this method is also limited by steric hindrance between antibodies.^78,79^ The image fusion approaches were hindered by autofluorescence and image noise, particularly when LA-ICP-MS resolution of 1 μm was used. This limited the utility of the image fusion approaches to larger spot sizes. These factors could be mitigated through the use of fluorescent dyes with higher quantum yields and thus lower excitation powers for the same emission intensity such as Alexa dyes^80^ or time-gated fluorescence imaging to reduce autofluorescent signal.^81^

## Conclusion

This study presents FRACTAL-MSI, a simple and flexible multimodal amplification strategy that combines iterative secondary antibody layering with dual fluorescent and metal labeling to address two persistent limitations in the use of elemental MSI to analyze biomolecules: insufficient sensitivity for low-abundance targets and the signal-to-resolution trade-off inherent to imaging at small spot sizes. By applying the approach to the analysis of NeuN, we showed that the method was applicable across fresh-frozen brain, cultured cells, and FFPE tissue with substantial increases in quantified ^169^Tm signal across the five generations of iterative secondary antibody application. Importantly, FRACTAL-MSI enabled detection of NeuN in FFPE samples where conventional immunolabeling often fails, suggesting that amplification can help recover signals from epitopes masked by fixation and embedding and may expand the accessible biomarker space in archival tissues.

The colocalized multimodal tags on a single section also unlock a new generation of quantitative, machine learning-free image fusion for LA-ICP-MS. PRIF and CDFIF enhanced spatial detail in 5 μm elemental images to the resolution of IF microscopy while retaining quantitative information. The benefit of fusion diminishes as elemental pixel sizes approach 1 μm as the resolutions of LA-ICP-MS and IF approach parity. Enhanced sensitivity further enabled nanoscale SRR with a 250 nm resolution, demonstrating a pathway for LA-ICP-MS to approach and, in some contexts, exceed the spatial scale of 40× IF. While SRR introduced increased noise, these results nevertheless underscore the value of amplification for pushing the performance envelope of existing instrumentation. Finally, proof-of-concept multiplexing indicates that FRACTAL-MSI can be integrated with primary-conjugated antibodies to selectively amplify key targets within multianalyte panels, though broader multiplex expansion will require careful host-species selection and may benefit from alternative mass analysers offering higher sensitivity at reduced dwell times.

Overall, FRACTAL-MSI provides an accessible, reagent-ready route to improved sensitivity and resolution in elemental bioimaging, enabling quantitative multimodal fusion and facilitating the detection of low-abundance biomarkers in challenging sample types. This workflow should be readily adaptable to other epitopes and imaging platforms, and it provides a strong foundation for future developments toward higher-plex, higher-resolution, and more clinically translatable MSI-based immunomapping.

## Method

### Reagents

1000 μg mL^–1^ Tm, Er and Nd standards and Seastar Baseline nitric acid (HNO_3_) were supplied by Choice Analytical (Thornleigh, New South Wales, Australia). FluorSave Reagent, Tris buffered saline (TBS), Tris base, ethylenediaminetetraacetic acid (EDTA), polyethylene glycol (Mn 400), gallic acid, ethanol and gelatin from porcine skin (type B) were purchased from Sigma-Aldrich (Castle Hill NSW, Australia). The Maxpar labeling kit was purchased from Standard BioTools (South San Francisco, CA, USA), and Active Motif MAX Stain Immunofluorescence Tools from Australian Biosearch (Wangara, WA, Australia). The list of antibodies used is provided in Table 2, and the antibodies with metal analytes were functionalized as per the Maxpar protocol. TrypLE Express Enzyme (×1 no phenol red), l-alanyl-l-glutamine dipeptide (GlutaMAX), fetal bovine serum (FBS) and Dulbecco’s Modified Eagle Medium (DMEM 5796) were purchased from Thermo Fisher Scientific (Scoresby VIC Australia). DMEM/F12 (D8062) and Phorbol 12-myristate 13-acetate (PMA) were purchased from Sigma (Castle Hill NSW, Australia).

**Table 1:** Scheme of G-Levels with Theoretical Antibody Quantities

G-Level	Fluorescent tag antibody layers	Additional antibodies per layer	MaxPar tag antibody layers	Additional antibodies per layer
G1	Rabbit anti-Donkey	2	Donkey anti-Rabbit	4
G2	2× Rabbit anti-Donkey	8	2× Donkey anti-Rabbit	16
G3	3× Rabbit anti-Donkey	32	3× Donkey anti-Rabbit	64
G4	4× Rabbit anti-Donkey	128	4× Donkey anti-Rabbit	256
G5	5× Rabbit anti-Donkey	512	5× Donkey anti-Rabbit	1024
Total	5× Rabbit anti-Donkey	682	5× Donkey anti-Rabbit	1364

**Table 2:** List of Antibodies and Their Respective Analyte

Antibody	Analyte	Code	Source
Rabbit monoclonal anti-NeuN antibody	None	ab177487	Abcam (Melbourne, Australia)
Rabbit anti-donkey IgG (H+L)	^169^Tm	SA1–26816	ThermoFisher (Brisbane, Australia)
Fluorescein (FITC) AffiniPure donkey anti-rabbit IgG (H+L)	FITC	711–095–152	Stratech Scientific APAC (Mona Vale, Australia)
Human MMP-11 antibody	^142^Nd	MAB3657	In-Vitro Technologies (Lane Cove West, Australia)
Human CD45 antibody	^170^Er	MAB1430	In-Vitro Technologies (Lane Cove West, Australia)

### Animal Welfare and Sampling

C57BL/6 healthy mice were housed at a maximum of 5 in a cage throughout the experiment. All mice were maintained on a 12 h light/dark cycle and had ad-libitum access to food and water. Room temperature was kept at a consistent 20–22 °C. Six-month-old mice were euthanized according to training protocol ETH20-5073, and the brains were then excised and snap frozen using dry ice. Frozen brains were cryo-sectioned at a thickness of 30 μm on a CryoStar NX70 Cryostat (Thermo Fischer; North Ryde, Australia) using a Tarsan-coated blade (UDT315 × 50; C. L. Sturkey, Inc.; Lebanon, PA).

Normal Sprague–Dawley rats were provided with standard rat chow and housed under a 12 h light/dark cycle with environmental enrichment. Animals were euthanized using lethal injection (pentobarbitone, 1 mL kg^–1^, i.p. [intraperitoneal]) and then subjected to cardiac perfusion with heparinized saline and 4% paraformaldehyde (PFA) as per ethics protocol ETH15-515. Formalin-fixed paraffin embedded (FPPE) rat coronal tissue blocks were prepared by immersion fixation in 10% NBF for 24 h and were subsequently paraffin embedded and sectioned at 5 μm on a Epredia HM 325 Rotary Microtome (Thermo Fisher Scientific Inc., Scoresby, VIC, Australia).

### Cell Culture

SH-SY5Y cells (passage 20-22) were maintained in T75 cell culture flasks in DMEM supplemented with 10% FBS and 2 mM GlutaMAX, and incubated in a humidified 37 °C, 5% CO_2_ environment. Upon reaching 80–85% confluency, cultures were incubated with TrypLE for 5 min at 37 °C, removed and centrifuged at 300*g* for 5 min at room temperature, before resuspension in the prepared DMEM and seeded at 1.8 × 105 cells mL^–1^ in T75 cell culture flasks.

To prepare the cells for imaging, they were seeded in two 12-well plates (one for untreated and one for differentiation) containing 15 mm sterile round glass coverslips at a density of 3.0 × 10^4^ cells mL^–1^ in DMEM and left to equilibrate for 24 h in a humidified 37 °C, 5% CO_2_ incubator. After 24 h in the differentiated plate, the DMEM was replaced with 0% FBS DMEM supplemented with 2 mM l-alanyl-l-glutamine and left to equilibrate for 24 h. The seeding media was replaced with fresh DMEM in the nondifferentiated plate. After this, the media in the differentiation plate was replaced with DMEM/F12 supplemented with 1% FBS, 2 mM l-alanyl-l-glutamine and 100 nM PMA. Cells were visually monitored for morphological change over the course of 5 days.

All cells were then washed three times in PBS and fixed with 2% paraformaldehyde at 4 °C overnight. Cells were washed with PBS again and excess aldehydes were quenched in 100 mM glycine for 5 min. Finally, the round coverslips were attached to a microscope slide cell side up with nail polish.

### Immunolabeling

Immunolabeling was modified from the original FRACTAL protocol^21^ and is briefly summarized here. Frozen samples were allowed to come to room temperature (RT) for 30 min and then fixed in 4% paraformaldehyde for 15 min. The tissue was washed in 1× TBS (3 × 5 min) and then blocked with MAXblock for 10 min at RT. Another 1× TBS (3 × 5 min) wash was performed before the primary anti-NeuN antibody (diluted to 5 μg mL^–1^ in MAXbind) was applied and incubated overnight at RT. A final 1× TBS (3 × 5 min) wash was performed before the application of secondary and tertiary antibodies.

After application of the primary antibody, the FITC-secondary donkey anti-rabbit antibody and the ^169^Tm-tertiary rabbit anti-donkey antibody were iteratively applied to the sample. All secondary and tertiary antibodies were diluted in MAXbind to 2.5 μg mL^–1^, before consecutive 20 min incubations in the dark and 1× TBS (2 × 5 min) washes up to the stated number of cycles (see Table 1). Levels of staining cycles are designated by their generation (G) level, with G1 equal to the application of a single pair of fluorophore and lanthanide-tagged antibodies (i.e., secondary and tertiary antibodies), and G5 containing 5 layers of the paired fluorophore and lanthanide-tagged antibodies (i.e., 10 total layers of secondary antibodies). The theoretical amplification for each level is shown in Table 1.

Finally, DAPI (0.1 μg·mL^–1^) and Cell-ID Intercalator-Ir (312.5 nM) were applied for 5 min each after a 1× TBS wash (3 × 5 min) with 0.2% triton-X. The slides were then coverslipped with FluorSave and left to harden overnight protected from light. The protocol was the same for all sample types apart from cells which did not undergo coverslipping and were imaged after dehydration with 100% ethanol washes (3 × 5 min). After IF imaging, the mounting media was removed using 1× TBS for 12 h, then were dehydrated with 100% ethanol washes (3 × 5 min). FFPE tissue sections were baked overnight at 37 °C, then deparaffinized and brough to water before washing and blocking for subsequent immunolabeling following the same protocol described above.

To assess the feasibility of including this protocol into a multiplexed analysis, monoclonal anti-MMP-11 was labeled with ^142^Nd and monoclonal anti-CD45 labeled with ^170^Er (Table 2). These antibodies were selected, as their clonality did not have cross-reactivity with the secondary antibodies used for amplification. The above protocol was followed until amplification of NeuN. Afterward, the nonamplified primary antibodies (anti-MMP-11, anti-CD45) were incubated for 12-h at RT before the remainder of the protocol was applied. Step-by-step instructions can be found in Table S2.

### Calibration Standards

External calibration standards were prepared from gelatin according to previously published protocols.^82,83^ Briefly, 5 mixed calibration solutions of Tm, Nd, Er and Ir and 1 blank were prepared in a buffer of 100 mM Tris–HCl (pH 7.4), 10 mM EDTA, 5 mg mL^–1^ gallic acid and 1% w/w polyethylene glycol in ultrapurified water (18.2 MΩ cm^–1^ at 25 °C, Arium Pro Vf, Sartorius, Goettingen, Germany). Six 10% gelatin solutions were created using the Tm, Nd, Er and Ir calibration standard solutions. The gelatin standard mixtures were heated to 38 °C until liquefied, vortexed for homogenization, and pipetted into a 6-well HybriWell on a standard microscope slide before prompt freezing at −80 °C for 15 min until opaque. Post-freezing, the Hybriwell mold was removed, and the gelatin standards were stored at room temperature until subjected to analysis. An 100 μL aliquot of each gelatin standard was dried, weighed, then dissolved in 1% HNO_3_ for final characterization with an Agilent 7700 ICP-MS (Agilent Technologies, Mulgrave, Australia) with a Micromist nebulizer and Scott type double pass spray chamber cooled to 2 °C for sample introduction. A 100 μg L^–1^ Rh solution in 1% HNO_3_ was used as an internal standard and introduced into the analyte flow via a T connector postpump. The element concentrations of each standard can be found in Table S3.

### LA-ICP-MS

LA-ICP-MS imaging was performed on an Elemental Scientific Lasers imageBIO 266 nm (Kennelec; Mitcham, Australia) equipped with a TwoVol3 ablation chamber and coupled to an Agilent 7900 series ICP-MS (Mulgrave, Australia) operated using the parameters in Table S4. The laser was operated with a fluence of 2 mJ cm^–1^, the repetition rate was set at 200 Hz for 1 μm spot size and 400 Hz for 5 μm spot size, with respective speeds of 20 or 100 μm s^–1^, and spot overlaps of 0.9 or 4.5 μm.^84^ Super-resolution reconstruction (SRR) was also applied to obtain images with submicron resolution.^74^ Briefly, the laser energy was adjusted to only ablate to half the tissue depth, and two orthogonal layers were ablated over the same area with a spot size of 1 μm and an overlap of 0.9 μm, a scan speed of 20 μm, and an offset between the two layers of 0.5 μm. The two layers are then summed together to create an image, smoothed with a Gaussian kernel, before a Richardson–Lucy Total Variance Regularisation and a Gaussian point spread function were applied. The resulting image achieved a resolution of 250 nm.

### Microscopy

Fluorescent images were taken using a ZEISS AxioScan Z.1 slide scanner (Carl Zeiss AG, Oberkochen, Germany). DAPI and FITC were visualized with excitation/emission wavelengths of 385/425 nm and 469/514 nm, respectively. Emission channel intensities were set to the same range across all fluorescent samples by using Zen 3.3 (Carl Zeiss AG, Oberkochen, Germany) before being exported as TIFFs.

### Data processing -LA-ICP-MS data processing and registration

LA-ICP-MS data was exported in the raw Agilent.b and iolite log.csv file formats into Pew^2^ ^85^ and visualized and quantified before further processing. LA-ICP-MS and IF micrographs were registered manually in Pew^2^ via a point set registration method, where 3 points were manually matched in each image to adjust for translation, rotation, and scaling, which was recorded as a transformation matrix.^86^ The transform matrix and the overlapping regions of LA-ICP-MS and IF images were exported as comma separated value files for downstream processing. SRR data were combined and processed with a custom python script adapted from the original MATLAB code.^74^

### Data Processing – Image Fusion

Two image fusion methods were tested on the multimodal data: the first via pixel redistribution image fusion (PRIF) and the second a cumulative density function image fusion (CDFIF). A custom Python script was then used to map between the LA-ICP-MS and IF pixels for both of the methods. For PRIF, all IF pixels contained within a single LA-ICP-MS pixel were normalized to have a mean value of 1 and then multiplied by the parent LA-ICP-MS pixel concentration. The result was a quantitative LA-ICP-MS map at the resolution of the IF image. The CDFIF image was obtained after the manually registered micrographs were used to convert all of the pixel values of the IF image to the quantified values of the LA-ICP-MS image. This was performed through normalizing the IF intensity histograms and cumulative distribution functions to their quantified LA-ICP-MS counterparts (see Figure S2). Image scale and calibration bars were added using FIJI^87^ for all of the imaging data.

## Supplementary Material



## Data Availability

All code used can be found in the https://github.com/davepbish/FRACTAL-MSI

## Notes

Tables S1–S4: Extended methods and instrument parameters. Figures S1–S7: Fluorescent microscopy, LA-ICP-MS and fused images of mono- and multiplex of murine brain and SH-SHSY5Y cells (PDF)
