# Supplementary material for: Multimodal FRACTAL-MSI and Image Fusion Improves Detection and Resolution of Biomolecule Imaging
Source: ACS Cent Sci. 2026 Jul 6;12(8):1119–31. doi: 10.1021/acscentsci.6c00422 (PMC13523496; doi:10.1021/acscentsci.6c00422)
Supplement: Supplementary file 1 [file oc6c00422_si_001.pdf]

Supplementary Information

Multimodal FRACTAL-MSI and image fusion improves detection and resolution of biomolecule imaging.

*Mika T. Westerhausen[1,2,3†], Rosemary J. Bergin[1,2†], Connor R. Phillips[2,4], Jake P. Violi[5], Thomas E. Lockwood[1,2,6], Reggie Ridlen[4], David P. Bishop[1,2]**

[1]School of Mathematical and Physical Sciences, University of Technology Sydney, Ultimo, NSW, 2007, Australia

[2]Hyphenated Mass Spectrometry Laboratory, University of Technology Sydney, Ultimo, NSW, 2007, Australia

[3]Molecular Horizons, University of Wollongong, Wollongong, NSW, 2500, Australia

[4]School of Life Sciences, Faculty of Science, University of Technology Sydney, Sydney, NSW, 2007, Australia

[5]School of Chemistry, University of New South Wales, Sydney, NSW, 2052, Australia

[6]NanoMicroLab, Institute of Chemistry, University of Graz, Graz, 38010, Austria

† These authors contributed equally

*email: david.bishop@uts.edu.au

Data availability statement: All MSI data and code is available at https://github.com/davepbish/FRACTAL-MSI

Funding: D.P.B. is supported by an Australian Research Council Discovery Project (DP230101740). R.J.B. is supported by the Australian Government Research Training Program Scholarship.

Conflict of interest disclosure: There are no conflicts of interest to declare.

Ethics approval statement: The animal experiments were performed with the approval of the University of Technology Animal Care and Ethics committee, ETH20-5073 and ETH15-515.

Patient consent statement: NA

Permission to reproduce material from other sources: NA

Clinical trial registration: NA

Keywords: Mass spectrometry imaging, LA-ICP-MS, signal amplification, multimodal imaging, image fusion

Table S1. Average counts per second raw signal obtained at the different G-levels from the samples in Figure 2.

G-Level	Average Counts per Second	Median Counts per Second
G1	10.39	9
G2	18.92	17
G3	40.56	39
G4	74.03	71.01
G5	87.39	84.01

Table S2. Step by step protocol of G1 to G5 staining of all sample types used.

Steps	Fresh frozen murine brain and cells	FFPE murine brain
**Pre immuno-labelling**	1. Fix in 4% paraformaldehyde for 15 mins<br>2. Wash in 1x TBS (3 x 5 mins)<br>3. Block with MAXblock™ for 10 minutes at RT<br>4. Wash in 1x TBS (3 x 5 mins)	1. Bake at 37 °C overnight, then deparaffinize with xylene<br>2. Sequential 90%, 70%, 50% ethanol solutions washes to bring samples to water<br>3. Wash in 1x TBS (3 x 5 mins)<br>4. Block with MAXblock™ for 10 minutes at RT
**Primary antibody immuno-labelling**	1. Apply primary anti-NeuN antibody (diluted to 5 μg mL^{-1} in MAXbind™) and incubate overnight at RT<br>2. Wash in 1x TBS (3 x 5 mins)	
**G1 immuno-labelling**	1. Apply secondary FITC- donkey anti-rabbit antibody (diluted in MAXbind™ to 2.5 μg mL^{-1}) and incubate for 20 min in the dark<br>2. Washed in 1x TBS (2 x 5 mins)<br>3. Apply tertiary ^{169}Tm-rabbit anti-donkey antibody (diluted in MAXbind™ to 2.5 μg mL^{-1}) and incubate for 20 min in the dark<br>4. Wash in 1x TBS (2 x 5 mins)	
**G2 immuno-labelling**	1. Apply secondary FITC- donkey anti-rabbit antibody (diluted in MAXbind™ to 2.5 μg mL^{-1}) and incubate for 20 min in the dark<br>2. Washed in 1x TBS (2 x 5 mins)<br>3. Apply tertiary ^{169}Tm-rabbit anti-donkey antibody (diluted in MAXbind™ to 2.5 μg mL^{-1}) and incubate for 20 min in the dark<br>4. Wash in 1x TBS (2 x 5 mins)	
**G3 immuno-labelling**	1. Apply secondary FITC- donkey anti-rabbit antibody (diluted in MAXbind™ to 2.5 μg mL^{-1}) and incubate for 20 min in the dark<br>2. Washed in 1x TBS (2 x 5 mins)	

	3. Apply tertiary ^{169}Tm-rabbit anti-donkey antibody (diluted in MAXbind™ to 2.5 µg mL^{-1}) and incubate for 20 min in the dark<br>4. Wash in 1x TBS (2 x 5 mins)
**G4 immuno-labelling**	1. Apply secondary FITC- donkey anti-rabbit antibody (diluted in MAXbind™ to 2.5 µg mL^{-1}) and incubate for 20 min in the dark<br>2. Washed in 1x TBS (2 x 5 mins)<br>3. Apply tertiary ^{169}Tm-rabbit anti-donkey antibody (diluted in MAXbind™ to 2.5 µg mL^{-1}) and incubate for 20 min in the dark<br>4. Wash in 1x TBS (2 x 5 mins)
**G5 immuno-labelling**	1. Apply secondary FITC- donkey anti-rabbit antibody (diluted in MAXbind™ to 2.5 µg mL^{-1}) and incubate for 20 min in the dark<br>2. Washed in 1x TBS (2 x 5 mins)<br>3. Apply tertiary ^{169}Tm-rabbit anti-donkey antibody (diluted in MAXbind™ to 2.5 µg mL^{-1}) and incubate for 20 min in the dark<br>4. Wash in 1x TBS (2 x 5 mins)
**Counter-staining**	1. Wash in 1x TBS wash (3 x 5 min) with 0.2% triton-X<br>2. Apply DAPI (0.1 µg.mL-1)) for 5 mins in the dark<br>3. Apply Cell-ID™ Intercalator-Ir (312.5 nM) for 5 mins in the dark

Table S3. Table of external standard concentrations and analytical performance.

Standards	^{169}Tm (mg kg^{-1})	^{193}Ir (mg kg^{-1})	^{142}Nd (mg kg^{-1})

1	0.00	0.27	1.69
2	51.49	0.54	6.18
3	105.73	0.90	10.40
4	182.88	1.66	19.04
5	287.70	1.75	40.03
6	616.07	3.44	72.69
LOD	0.13	0.01	0.45
R^2	0.9969	0.9828	0.9836

Table S4. ICP-MS parameters.

Laser MFC 1 and 2	0.5, 0.2 mL min^{-1} He
RF Power	1350 W
Carrier gas flow	0.45 L min^{-1}
Sample depth	5 mm
Extract lens 1 and 2	-2 V, -200 V
Omega bias and lens	-115 V, 7.5 V
Octopole bias and RF	-20V, 200V
Deflect	5 V
Collision gas	3 mL min^{-1} H_2

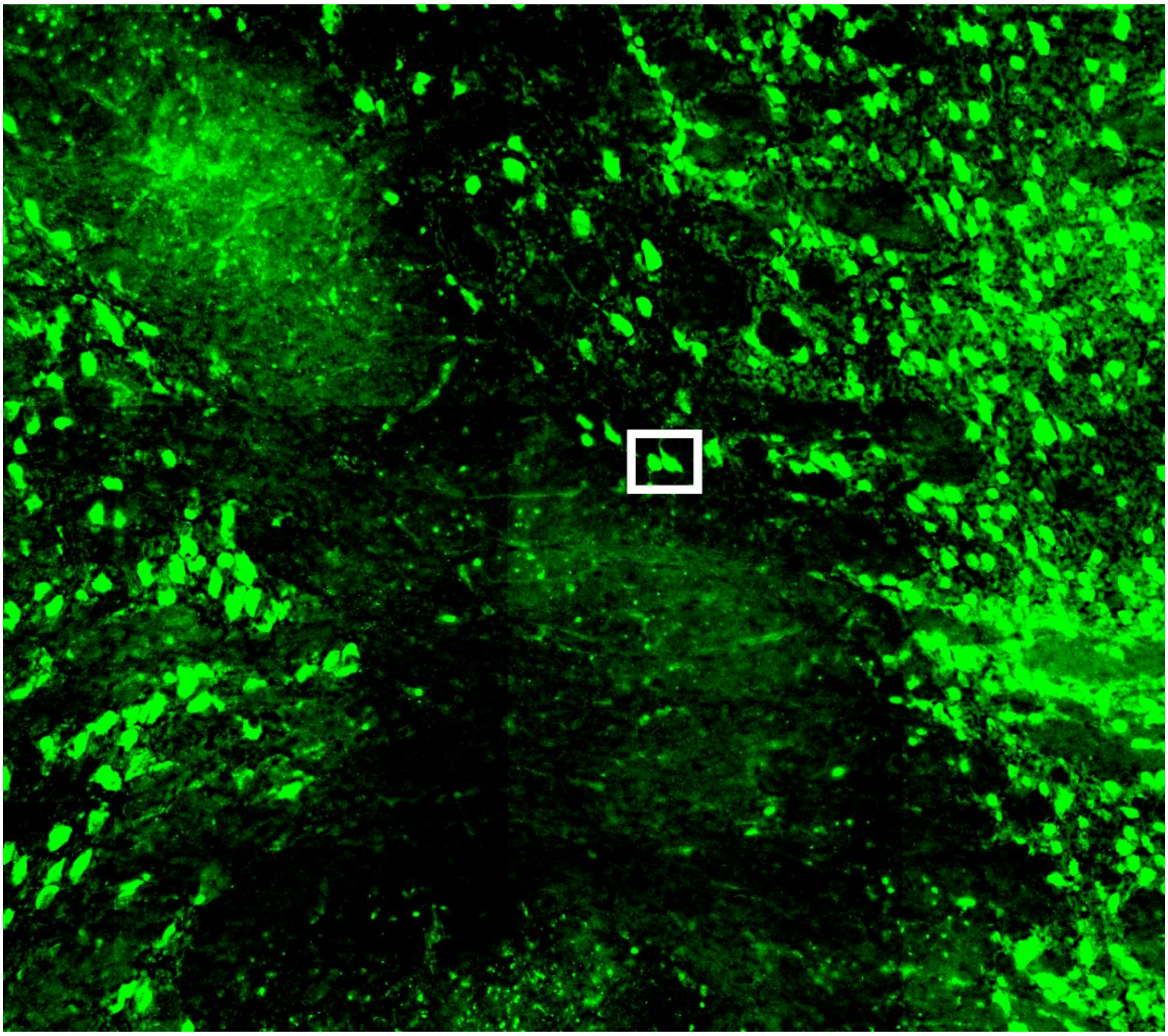

Figure S1. Corresponding 40x IF image of Figure 3a with region of interest highlighted with a white box.

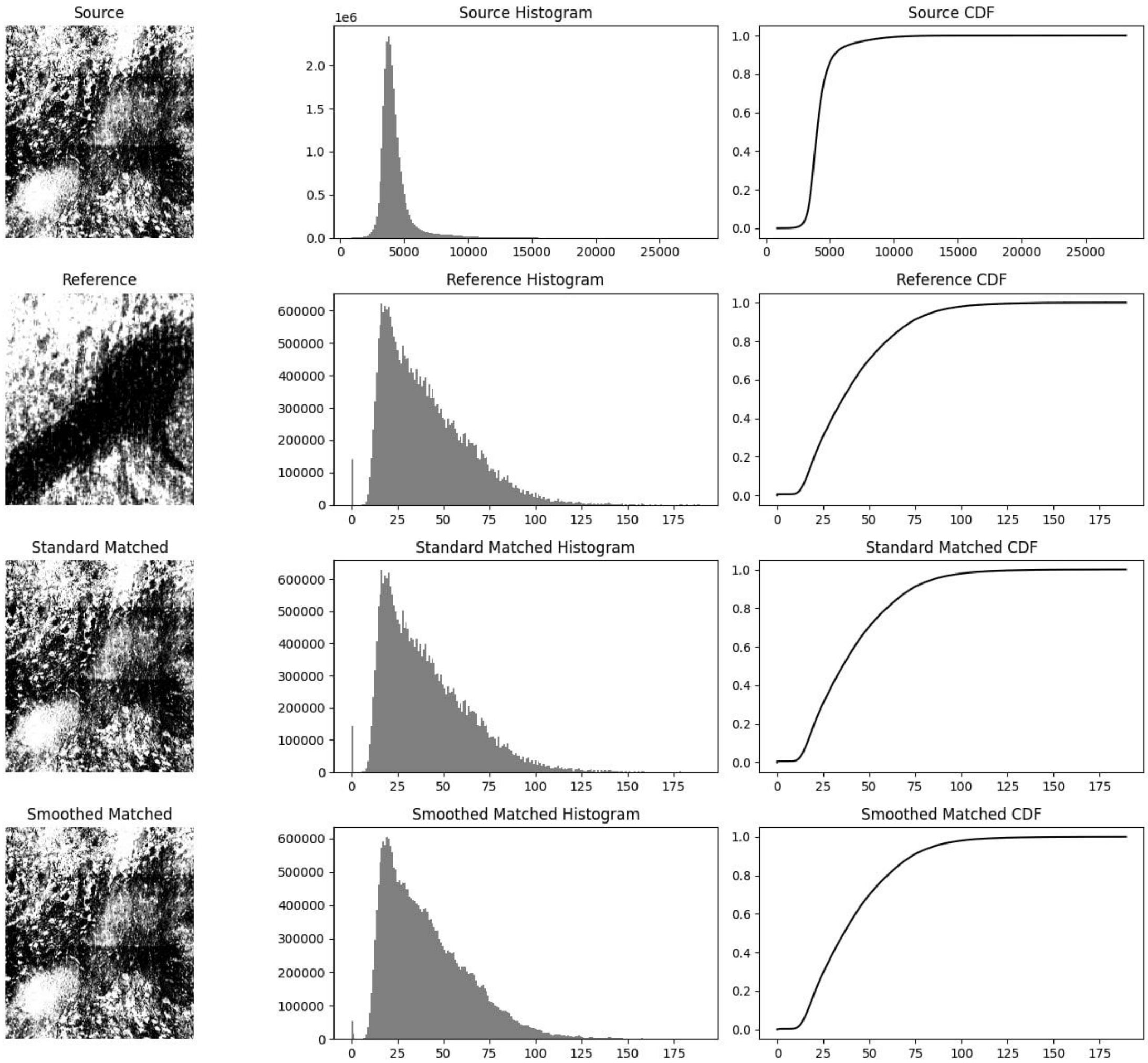

Figure S2. Histogram progression of CDFIF method using the source (IF image) and reference (LA-ICP-MS image). Step by step: from the histogram of the source image (IF microscopy image) and reference image (LA-ICP-MS image) cumulative density functions (CDF) are derived, next the source CDF is matched to the reference CDF to redistribute the original signal intensity to the intensity distribution of the reference image, and finally the histogram is smoothed to remove artefacts from CDF matching.

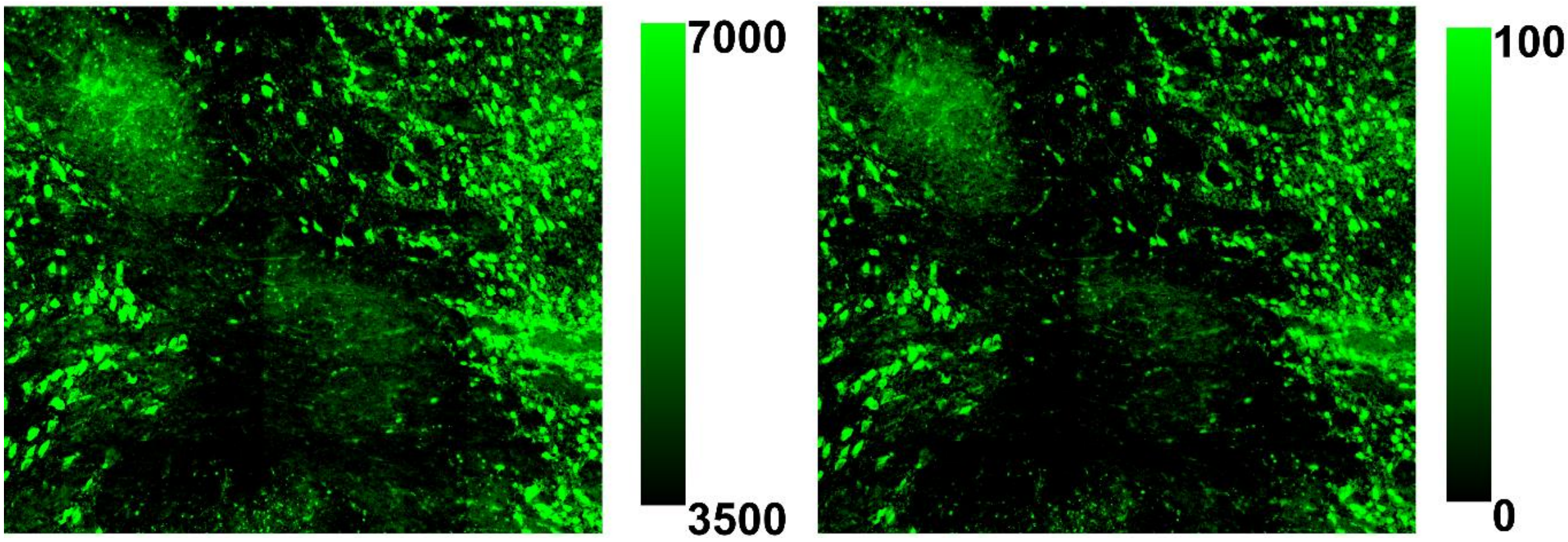

Figure S3. Comparison of 40x magnification immunofluorescence image (left) versus CDF image fusion on 5µm LA-ICP-MS (right). The images are identical with only their intensity scales differing. Calibration bars are in raw intensity units for IF and mg kg^{-1} for CDFIF.

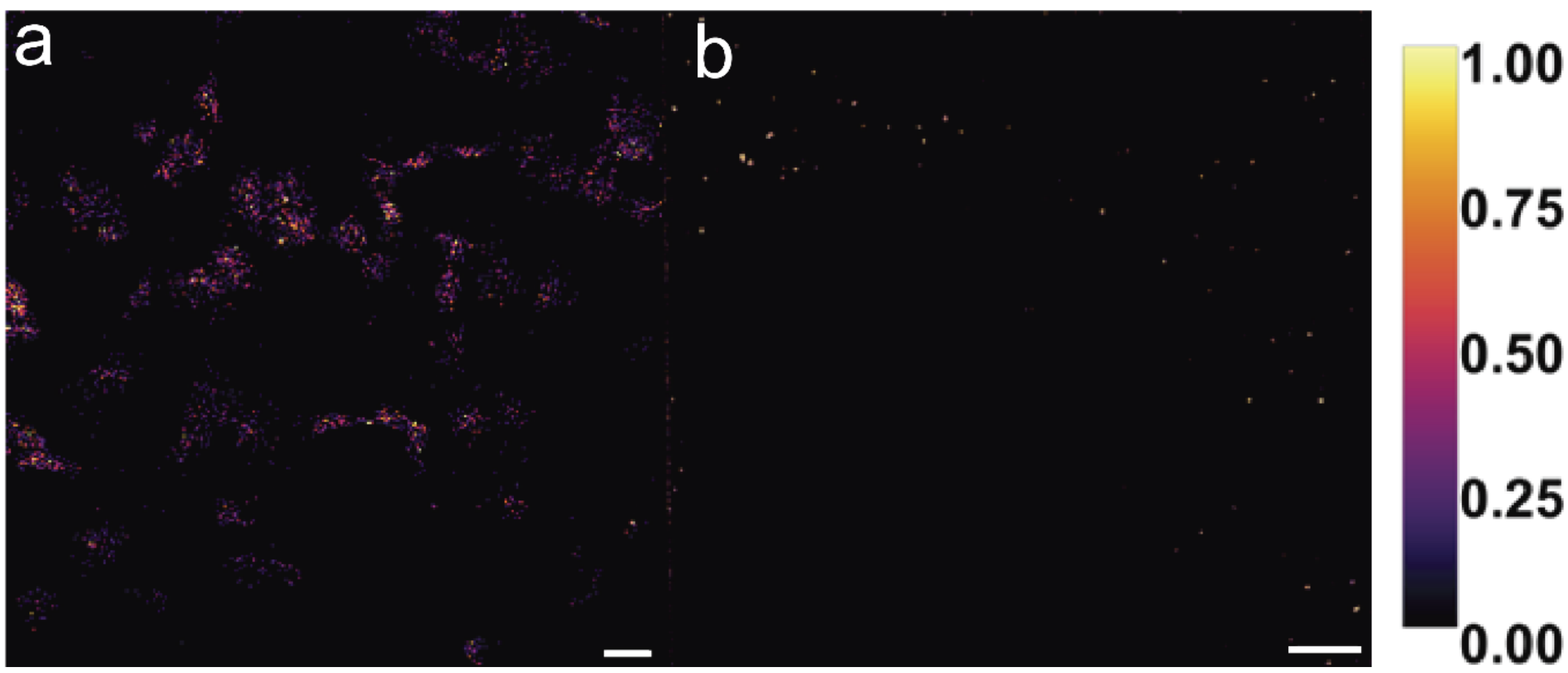

Figure S4. G1 anti-NeuN ^{169}Tm staining on a) SHSY5Y cells and b) FFPE murine brain. Scale bar is 10 µm for a) and 100 µm for b) with calibration bar in mg kg^{-1}.

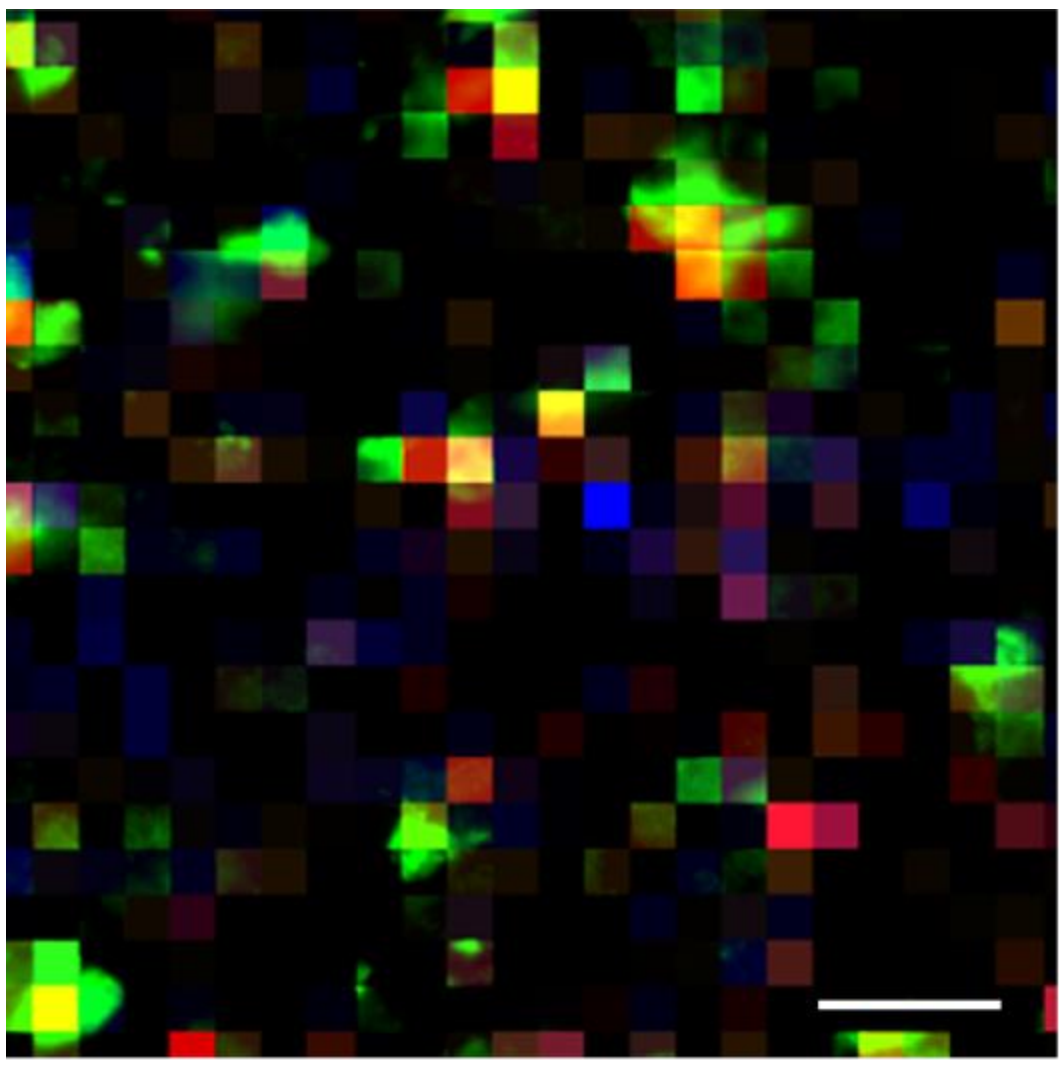

Figure S5. Multiplex false colour image of the nucleus (^{193}Ir intercalator, blue), PRIF NeuN (^{169}Tm, green), CD45 (^{170}Er, red) and MMP-11 (^{142}Nd, Orange). Scale bar is 20 μm.

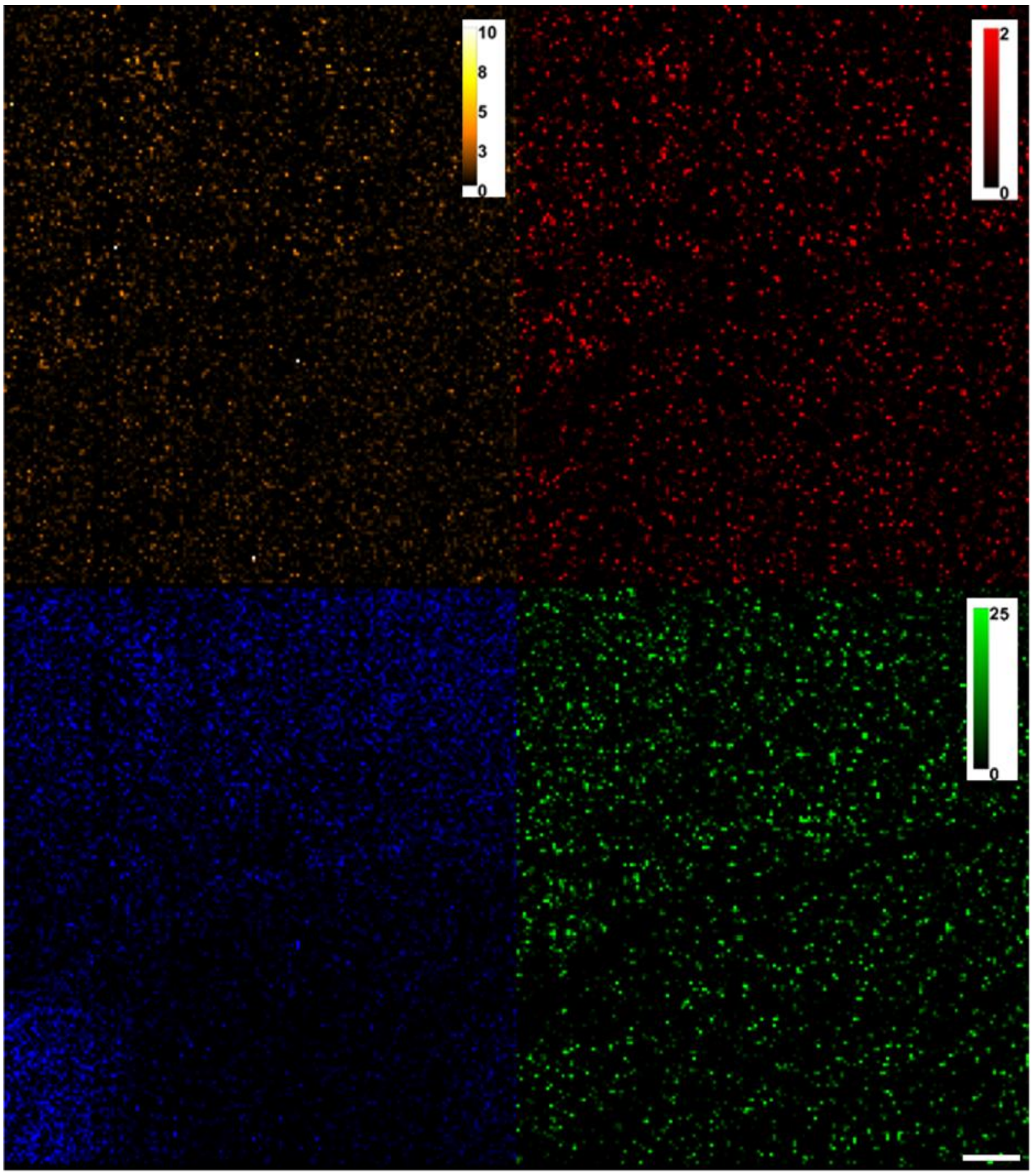

Figure S6. The individual elemental channels from the 5 μm LA-ICP-MS multiplex. Clockwise from the top-left MMP-11 (^{142}Nd, Orange) , CD45 (^{170}Er, red), NeuN (^{169}Tm, green) and nuclear stain (^{193}Ir intercalator, blue). Scale bar is 100 μm.

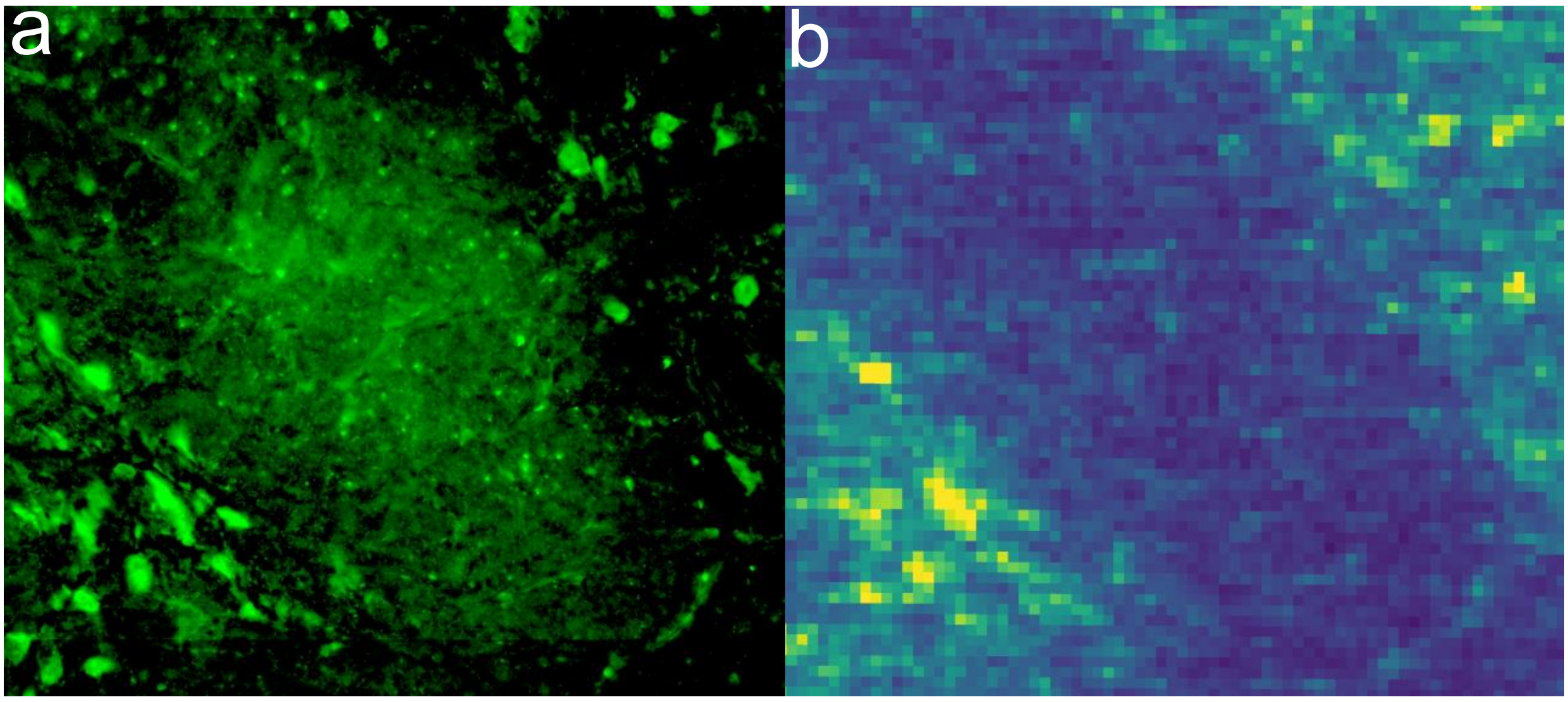

Figure S7. Comparison of localized imaging artefacts of a) autofluorescence in an IF image of mouse brain and b) blurred pixel in the line scan direction of mouse brain from 5µm LA-ICP-MS.
